# Oviposition selection in spotted lanternfly: impact of habitat and substrate on egg mass size and hatchability

**DOI:** 10.3389/finsc.2022.932433

**Published:** 2022-07-28

**Authors:** Houping Liu

**Affiliations:** Pennsylvania Department of Conservation and Natural Resources, Harrisburg, PA, United States

**Keywords:** *Lycorma delicatula*, egg mass structure, hatch success, Fulgoridae, invasive species

## Abstract

Oviposition strategies adopted by insects (e.g., habitat selection, substrate preference, egg size, clutch size, structure, arrangement, parental care) are critical to the survival and development of their eggs. The impact of habitat and oviposition substrate on spotted lanternfly egg mass size and hatchability was studied in Pennsylvania through laboratory observations and field monitoring in 2019 and 2021. Eggs were arranged in single layers of 1–13 columns (1–18 eggs/column) on surfaces of various types of oviposition substrates, with the longest column(s) in the middle of the egg mass. Egg mass size was positively correlated with column number, with a mean of 26.6–35.1 (0–105) eggs/egg mass for different samples. Significant differences in egg mass size were observed between study sites, with larger egg masses found at Wertz (44.8), Sam Lewis (40.6), Pinnacle (39.1), Marsh Creek (37.9), Susquehannock (34.5), and Memorial Lake (33.3) and smaller egg masses at Nolde Forest (25.0), Gordon (24.4), and Antietam (21.0). Significant differences were also detected between types of oviposition substrates with smaller egg masses found on American hornbeam (22.7). In general, more (31.6%–48.0%) eggs hatched in the field compared with the laboratory (10.0%). Egg hatch success was positively correlated with egg mass size, with the highest rates recorded on American beech, American hophornbeam, black birch, black cherry, black locust, hackberry, Norway maple, red maple, and sweet cherry at Wertz, Marsh Creek, Memorial Lake, and Pinnacle. Potential (positive or negative) impacts of tree-of-heaven density, initial infestation, treatment history, and incubation conditions are discussed.

## Introduction

Insect eggs are vulnerable to mortality factors such as parasitoids, predators, pathogens, and unfavorable weather conditions in the field (1). Eggshells, oviposition sites, maternal secretions, and other built-in defense mechanisms provide protection to eggs in various environments (1–4). As a life stage which cannot actively defend itself, the escaping strategies for insect eggs include parental care, sociality, concealment, and egg mass formation (1, 5). For example, thick spumaline coating protects egg masses of some caddisfly species from predation by *Orthotrichia armata* Wells (Trichoptera: Hydroptilidae) larvae (6), whereas egg-stacking and scale-casing are used by *Lymantria dispar* (L.) (Lepidoptera: Erebidae) and *Ochrogaster lunifer* Herrich-Schäffer (Lepidoptera: Notodontidae) to prevent inner layer eggs from being parasitized by certain species (7–10). Oviposition strategies have profound impacts on egg survival.

The spotted lanternfly, *Lycorma delicatula* (White) (Hemiptera: Fulgoridae), a univoltine pest of tree-of-heaven (*Ailanthus altissima* (Mill.) Swingle [Sapindales: Simaroubaceae]) from China (11), was introduced to Berks County, Pennsylvania, in 2014 (12, 13). It is currently found in 11 states from Massachusetts to Indiana in eastern United States (14). Egg masses are laid on various types of substrates (e.g., surfaces of trees, shrubs, vines, stones, fence posts, and other non-living materials) from mid-September to early November in Pennsylvania, with American beech (*Fagus grandifolia* Ehrh. [Fagales: Fagaceae]), black birch (*Betula lenta* L. [Fagales: Betulaceae]), black cherry (*Prunus serotina* Ehrh. [Rosales: Rosaceae]), grapes (*Vitis* spp. [Vitales: Vitaceae]), Norway maple (*Acer platanoides* L. [Sapindales: Sapindaceae]), red maple (*Acer rubrum* L. [Sapindales: Sapindaceae]), sweet cherry (*Prunus avium* L. [Rosales: Rosaceae]), tree-of-heaven, and tuliptree (*Liriodendron tulipifera* L. [Magnoliales: Magnoliaceae]) as favorites (15–17).

The size and hatch success of *L. delicatula* egg masses have been reported in its native range of Asia (11, 18–20). Limited observations on egg mass characteristics and comparative hatching in the laboratory and the field were also carried out in North America as parts of related studies (16, 21). However, systematic studies in egg mass structure, size, and hatchability are still lacking for the better understanding of their impacts on *L. delicatula* population dynamics in the field. It is hypothesized that habitat and substrate play an important role in the oviposition selection of *L. delicatula*. The objectives of this study were therefore to 1) understand the basic structure of *L. delicatula* egg masses, 2) examine the impact of habitat and oviposition substrate on egg mass size, and 3) compare egg hatch success in the laboratory and the field.

## Materials and methods

### Study sites

This study was carried out at 11 mixed hardwood sites (~0.5 ha) in Pennsylvania between 16 and 80 km South and West of the initial introduction in Berks County. See **Table 1** for location, type, structure, tree-of-heaven density, year of infestation, and treatment history for each study site. Study sites were at least 5 km apart from each other except Gibraltar North and Gibraltar South which were on the opposite sides of the same mountain ridge. Site Antietam was used in both 2019 and 2021. The number of tree-of-heaven trees with a diameter at breast height (DBH) >5 cm was recorded in each study site at the start of the field work. Northern spicebush (*Lindera benzoin* L. [Laurales: Lauraceae]) and summer grape (*Vitis aestivalis* Michx. [Vitales: Vitaceae]) were the most common understory species at all study sites.

**Table 1:** Study site location, type, structure, tree-of-heaven density, year of infestation, and treatment history in 2019 and 2021.

Name	Latitude Longitude	Type	Structure	Tree-of-heaven density^a^	Year of infestation	Chemical treatment^b^	Herbicide treatment^c^
***2019***							
Antietam	40.35086 -75.87749	County park	South-facing upper slope dominated by mature black birch and black cherry	Low	2018	No	No
Gibraltar North	40.28670 -75.88703	State forest	North-facing middle slope dominated by mature black birch and tuliptree	Low	2018	Yes	Yes
Gibraltar South	40.28697 -75.89678	State forest	South-facing middle slope dominated by mature black birch and American beech	Low	2018	No	No
Marsh Creek	40.06542 -75.73059	State park	Level lakeside dominated by mature Norway maple and black cherry	High	2018	No	No
Nolde Forest	40.27140 -75.94782	State park	East-facing lower slope dominated by mature black locust and young black cherry	Low	2018	Yes	Yes
Wertz	40.31869 -76.11358	State forest	East-facing upper slope dominated by mature black birch and American beech	Medium	2018	No	No
***2021***							
Antietam	40.35086 -75.87749	County park	South-facing upper slope dominated by mature black birch and black cherry	Low	2018	No	No
Gordon	39.93546 -75.59956	Nature area	West-facing upper slope site dominated by mature red maple and white ash	Low	2018	No	No
Memorial Lake	40.41705 -76.59359	State park	Level lakeside dominated by young red maple and white ash	High	2020	No	No
Pinnacle	39.84534 -76.34342	State park	West-facing upper slope dominated by young black birch and red maple	Medium	2020	No	Yes
Sam Lewis	39.99316 -76.54415	State park	East-facing middle slope dominated by mature eastern white pine and red maple	Medium	2020	No	No
Susquehannock	39.80538 -76.28144	State park	East-facing upper slope dominated by mature hackberry and young pawpaw	Medium	2020	No	Yes

a By total number of tree-of-heaven trees (>5 cm in diameter) on site—low: <20, medium: 21~50, high: >50.

b Dinotefuran trunk spray on tree-of-heaven for L. delicatula control in adjacent areas in 2018.

c Triclopyr or glyphosate trunk hack-and-squirt for tree-of-heaven control in adjacent areas in 2018 (Gibraltar North and Nolde Forest) and 2020 (Pinnacle and Susquehannock).

### Egg mass collection

**Figure 1:**
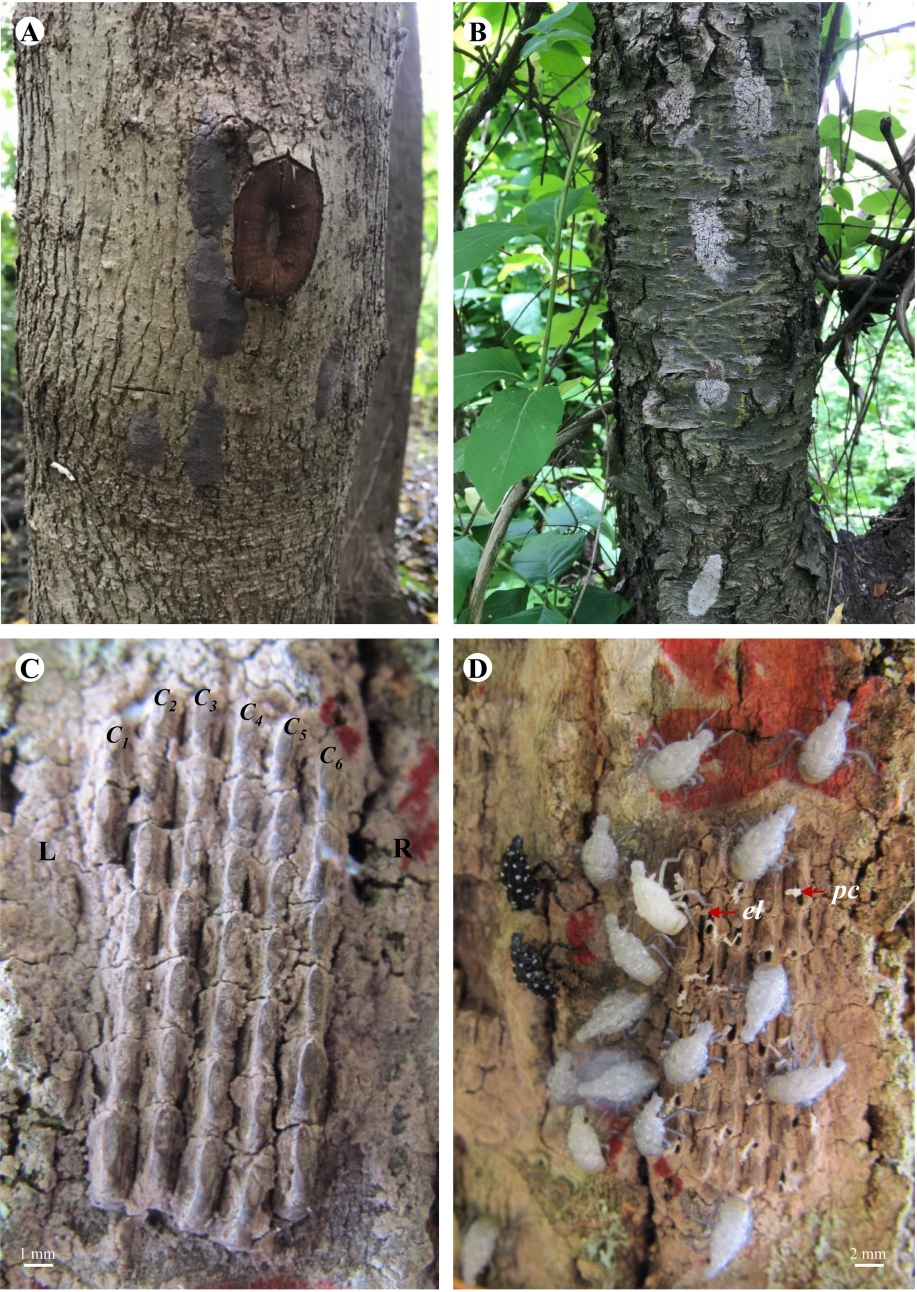
*Lycorma delicatula* egg masses in the field. **(A)** Newly laid on red maple. **(B)** Overwintered on black cherry. **(C)** Arrangement. **(D)** Hatching. L—left, R—right, *C_1_*—column #1, *C_2_*—column #2, C*_3_*—column #3, C*_4_*—column #4, *C_5_*—column #5, C*_6_*—column #6, *el*—egg lid, *pc*—pronymphal cuticle.

Egg mass collection was carried out at six study sites (Antietam, Gibraltar North, Gibraltar South, Nolde Forest, Marsh Creek, and Wertz) in late April 2019. *Lycorma delicatula* egg masses are dark gray in color at the beginning (**Figure 1A**) and turn grayish white the next spring (**Figure 1B**). At each study site, the surfaces of live trees, shrubs, and vines were searched for *L. delicatula* egg masses. Egg masses found on the lower 2-m trunk of the tree (shrub/vine) were collected using a 1.27-cm bench chisel (Buck Brothers, Everett, WA). A rectangle was created first to surround the egg mass by cutting directly into the bark at 0.5 cm away from its outer margins. The egg mass on the surface was then dislodged by gently pushing the chisel under the bark rectangle upward from the lower end. Care was taken to ensure no eggs were accidentally missed, cut, squeezed, or otherwise damaged. Each dislodged egg mass was then held in a 50-ml centrifuge tube (VWR International, Radnor, PA) and labeled by collection date, study site, and type of oviposition substrate before being brought back to the laboratory for examination and incubation.

### Egg mass marking in the field

Egg mass marking was performed at Antietam, Gibraltar North, Gibraltar South, Nolde Forest, Marsh Creek, and Wertz in 2019 and Antietam, Gordon, Memorial Lake, Pinnacle, Sam Lewis, and Susquehannock in 2021. Current generation egg masses found on the lower 2-m trunk of different types of oviposition substrates near the epicenter at each study site were circled with a yellow timber crayon (Dixon Ticonderoga, Heathrow, FL) in late April for monitoring.

### Egg mass structure

*Lycorma delicatula* eggs are cylindrical with a diameter of 1.5 mm and height of 3.0 mm. They are usually laid in single layer masses with 5–10 columns (10–30 eggs/column) and covered by a layer of gray wax in Asia (11). To characterize the structure of the *L. delicatula* egg masses in North America, column number (**Figure 1C**) was recorded from left to right for egg masses collected in 2019 and those marked in the field in 2021, whereas egg number was counted for all egg masses (collected and marked) before hatch. All egg masses were counted again within 2 weeks after hatch completed to ensure accuracy. Direct count was possible for egg masses with little or no waxy cover on the top; however, for those with a thick waxy cover, wax removal with a #2 camel hairbrush (Grumbacher, Leeds, MA) was needed for exact enumeration. This procedure was only carried out after hatch when necessary to avoid potential influence on hatch success. The numbers of eggs and numbers of columns for each egg mass were categorized at intervals of 10 and 1, respectively. Size category, column category, and column size (no. eggs/column) were used in data analysis. Travel restrictions stemming from the COVID-19 pandemic forced cancellations of scheduled field works in 2020 and early 2021.

### Egg hatch in the laboratory

Egg masses collected in 2019 were brought back to the laboratory for incubation inside a Percival incubator (model #DR-36VL, Percival Scientific, Perry, IA) at 22 ± 1°C, 40 ± 5% relative humidity (RH), and a 16:8-h photoperiod (light: dark) for 8 weeks (16). Egg mass and egg hatch success were monitored with the number of newly hatched nymphs recorded and removed weekly. Egg mass hatch success was calculated by dividing the number of egg masses with at least one hatched egg by the total number of egg masses at the study site, whereas egg hatch success was calculated by dividing the number of hatched eggs by the total number of eggs in the egg mass.

### Egg hatch in the field

Egg hatch in the field was monitored weekly on the marked egg masses for 8 weeks from mid-May to early July in 2019 and 2021. Presence of white 1st-instar nymphs or open egg lids (opercula) and attached whitish/yellowish pronymphal cuticles (**Figure 1D**) indicates hatch success (20, 22). Hatch success was calculated as described before.

### Data analysis

Data analysis was carried out in R (Version 3.3.3) (23). Egg counts per egg mass for field-collected and marked eggs in both years were subjected to Shapiro–Wilk normality test before analysis. If data were overdispersed, a negative binomial generalized linear (nbGLM in R) was used to detect the effect of study site or type of oviposition substrate. Kruskal–Wallis test was used to separate different columns based on eggs per column for egg masses collected or marked in the field. Significant effects were followed by pairwise Wilcoxon rank-sum test with a *P*-value adjusted by Benjamini–Hochberg method (24). A generalized linear model (GLM) with binomial distribution was used to examine the frequency of size category, frequency of column category, correlation between egg hatch success and egg mass size, or effect of study site or type of oviposition substrate on egg hatch success in each year. A generalized linear model was used to fit egg number with column number in each year.

## Results

In total, 300 egg masses were collected from 19 types of oviposition substrates at six study sites (50 egg masses/study site) in 2019, with most found on black birch, red maple, Norway maple, sweet cherry, tuliptree, black locust (*Robinia pseudoacacia* L. [Fables: Fabaceae]), tree-of-heaven, and black cherry (**Table 2**). Egg masses were also found on red oak (*Quercus rubra* L. [Fagales: Fagaceae]), black walnut (*Juglans nigra* L. [Fagales: Juglandaceae]), black willow (*Salix nigra* Marshall [Malpighiales: Salicaceae]), American hophornbeam (*Ostrya virginiana* (Mill.) K. Koch [Fagales: Betulaceae]), autumn olive (*Elaeagnus umbellata* Thunb. [Rosales: Elaeagnaceae]), northern spicebush, princess tree (*Paulownia tomentosa* (Thunb.) Steud [Lamiales: Paulowniaceae]), American beech, sassafras (*Sassafras albidum* (Nutt.) Nees [Laurales: Lauraceae]), shagbark hickory (*Carya ovata* (Mill.) K. Koch [Fagales: Juglandaceae]), and summer grape (**Table 2**).

**Table 2:** *Lycorma delicatula* egg mass collection by study site and oviposition substrate in 2019.

Substrate	Code	AT	GN	GS	MC	NF	WZ	Sub
*Acer platanoides*	Nm	1			32			33
*Acer rubrum*	Rm	4	2		18	6	25	55
*Ailanthus altissima*	Toh		4	4		4		12
*Betula lenta*	Bb	43	29	30				102
*Carya ovata*	Shag		1					1
*Elaeagnus umbellata*	Ao					4		4
*Fagus grandifolia*	Ab			1				1
*Juglans nigra*	Bw		3			4		7
*Lindera benzoin*	Spb			2				2
*Liriodendron tulipifera*	Tt		2	6		6		14
*Ostrya virginiana*	Hhb						4	4
*Paulownia tomentosa*	Pau		2					2
*Prunus avium*	Sc	1					21	22
*Prunus serotina*	Bc		3	1		6		10
*Quercus rubra*	Ro		3	6				9
*Robinia pseudoacacia*	Bl					13		13
*Salix nigra*	Wil					7		7
*Sassafras albidum*	Sas	1						1
*Vitis aestivalis*	Grp		1					1
**Total**		50	50	50	50	50	50	**300**

AT, Antietam; GN, Gibraltar North; GS, Gibraltar South; MC, Marsh Creek; NF, Nolde Forest; WZ, Wertz; Ab, American beech; Ao, autumn olive; Bb, black birch; Bc, black cherry; Bl, black locust; Bw, black walnut; Grp, summer grape; Hhb, American hophornbeam; Nm, Norway maple; Pau, princess tree; Rm, red maple; Ro, red oak; Sas, Sassafras; Sc, sweet cherry; Shag, Shagbark hickory; Spb, northern spicebush; Toh, tree-of-heaven; Tt, tuliptree; Wil, black willow.

In addition, 212 egg masses were marked in the field, including 120 egg masses on four types of oviposition substrates at six study sites (20 egg masses/study site, two types of oviposition substrates/study site, five plants/type of oviposition substrate, two egg masses/plant) in 2019, and 92 egg masses on 10 types of oviposition substrates at six study sites (4–20 egg masses/study site, 1–4 types of oviposition substrates/study site, 1–5 plants/type of oviposition substrate, 1–5 egg masses/plant) in 2021 (**Table 3**). The low population density at Antietam and Gordon in 2021 prevented more egg masses from being marked at those study sites. Tree-of-heaven was represented at all study sites in both years except Gordon in 2021, whereas black birch, black locust, and Norway maple were used in 2019, and American hornbeam (*Caprinus caroliniana* Walter [Fagales: Betulaceae]), black birch, black cherry, boxelder (*Acer negundo* L. [Sapindales: Sapindaceae]), hackberry (*Celtis occidentalis* L. [Rosales: Cannabaceae]), pawpaw (*Asimina triloba* (L.) Dunal [Magnoliales: Annonaceae]), red maple, shagbark hickory, and white ash (*Fraxinus americana* L. [Lamiales: Oleaceae]) were used in 2021 (**Table 3**).

**Table 3:** *Lycorma delicatula* egg mass marking in the field by year, study site, and oviposition substrate.

Substrate	Code	2019							2021						
		AT	GN	GS	MC	NF	WZ	Sub	AT	GD	ML	PC	SL	SQ	Sub
*Acer negundo*	Box													5	5
*Acer platanoides*	Nm				10			10							
*Acer rubrum*	Rm										5	5	5		15
*Ailanthus altissima*	Toh	10	10	10	10	10	10	60	4		5	5	5	5	24
*Asimina triloba*	Paw												5	5	10
*Betula lenta*	Bb	10	10	10			10	40				5			5
*Carpinus caroliniana*	Ahb									3					3
*Carya ovata*	Shag									5					5
*Celtis occidentalis*	Hack													5	5
*Fraxinus americana*	Wash										5				5
*Prunus serotina*	Bc										5	5	5		15
*Robinia pseudoacacia*	Bl					10		10							
**Total**		20	20	20	20	20	20	**120**	4	8	20	20	20	20	**92**

AT, Antietam; GD, Gordon; GN, Gibraltar North; GS, Gibraltar South; MC, Marsh Creek; ML, Memorial Lake; NF, Nolde Forest; PC, Pinnacle; SL, Sam Lewis; SQ, Susquehannock; WZ, Wertz; Ahb, American hornbeam; Bb, black birch; Bc, black cherry; Bl, black locust; Box, boxelder; Hack, hackberry; Nm, Norway maple; Paw, pawpaw; Rm, red maple; Shag, shagbark hickory; Toh, tree-of-heaven; Wash, white ash.

### Egg mass structure

**Figure 2:**
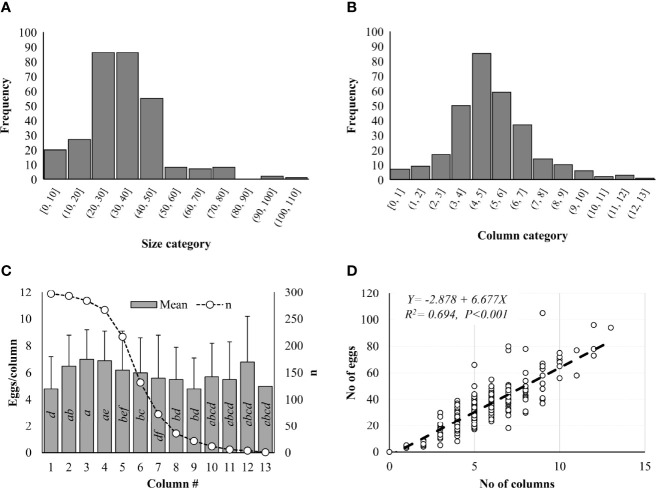
*Lycorma delicatula* egg mass structure in 2019. **(A)** Size category. **(B)** Column category. **(C)** Mean egg number per column. **(D)** Correlation between egg number and column number as indicated by the dash line. Means with the same lowercase letters are not significantly different (Wilcoxon rank-sum test, α = 0.05).

Number of eggs in each *L. delicatula* egg mass ranged from 0 to 105 in Pennsylvania based on 300 egg masses collected from the field in 2019 (**Figure 2A**). Significant differences in frequency were observed among different size categories (Z-value = -11.820, *P* < 0.001). Three egg masses (one from Gibraltar North and two from Nolde Forest) contained no eggs. Most egg masses (91.3%) contained <50 eggs, 75.6% had 20–50 eggs, and 15.7% had <20 eggs/egg mass (**Figure 2A**). Only 1 egg mass had >100 eggs while 2 had >90, 8 with >70, 7 had >60, and 8 had >50 eggs (**Figure 2A**). Significant differences in frequency were also observed among different column categories (Z-value = -7.141, *P* < 0.001). Eggs were arranged in 1–13 columns within the egg masses. Most egg masses (88%) contained <7 columns while 77% had 3–7 columns and 11% had <3 columns (**Figure 2B**). One egg mass had 13 columns while 3 had 12, 2 had 11, 6 had 10, 10 had 9, and 14 had 8 columns (**Figure 2B**). In total, 10,115 eggs arranged in 1,644 columns were recorded from 297 egg masses with at least 1 egg, including 1,601 eggs (270 columns) at Antietam, 1,447 eggs (239 columns) at Gibraltar North, 1,683 eggs (288 columns) at Gibraltar South, 1,249 eggs (225 columns) at Nolde Forest, 1,893 eggs (286 columns) at Marsh Creek, and 2,242 eggs (336 columns) at Wertz. Column size ranged from 1 to 18 with a mean of 6.2 ± 2.6 eggs/column and differed significantly between columns (χ^2^ = 179.610, df = 12, *P* < 0.001). Significant differences were found between columns 2 and 1, 7; 3 and 1, 5, 6, 7, 8, 9; and 4 and 1, 6, 7, 8, 9 (**Figure 2C**). A significant positive correlation was found between column number and egg number for the egg masses (F = 676.700, df = 1, 298, *P* < 0.001) (**Figure 2D**), with total eggs in each egg mass increasing with the increase of columns in it.

**Figure 3:**
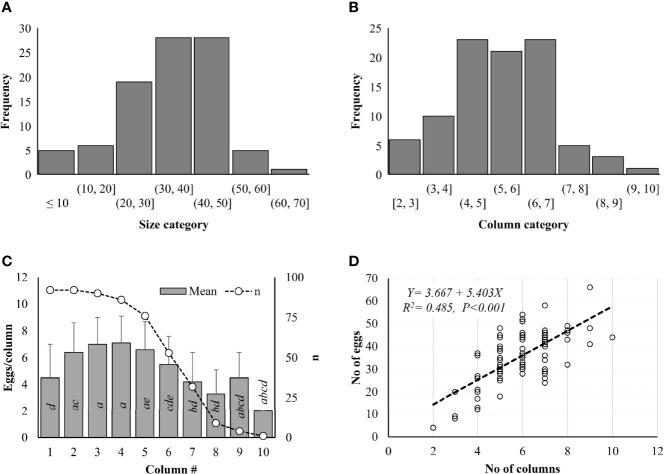
*Lycorma delicatula* egg mass structure in 2021. **(A)** Size category. **(B)** Column category. **(C)** Mean egg number per column. **D)** Correlation between egg number and column number as indicated by the dash line. Means with the same lowercase letters are not significantly different (Wilcoxon rank-sum test, α = 0.05).

*Lycorma delicatula* egg mass size ranged from 4 to 66 in Pennsylvania based on 92 egg masses marked in the field in 2021 (**Figure 3A**). No significant difference in frequency was observed among different size categories (Z-value = -0.448, *P* = 0.654). Most egg masses (93.5%) contained <50 eggs, 81.5% had 20–50 eggs, and 12.0% had <20 eggs/egg mass (**Figure 3A**). Only one egg mass contained >60 eggs while five had >50 eggs (**Figure 3A**). No significant difference in frequency was observed among different column categories either (Z-value = 0.932, *P* = 0.351). Eggs were arranged in 1–10 columns within the egg masses. Most egg masses (90.2%) contained <7 columns, while no egg masses contained <2 columns (**Figure 3B**). One egg mass had 10 columns while 3 had 9 and 5 had 8 columns (**Figure 3B**). In total, 3,288 eggs in 535 columns were counted from 92 egg masses, including 84 eggs (16 columns) at Antietam, 195 eggs (39 columns) at Gordon, 666 eggs (108 columns) at Memorial Lake, 781 eggs (132 columns) at Pinnacle, 690 eggs (118 columns) at Sam Lewis, and 812 eggs (122 columns) at Susquehannock. Column size ranged from 1 to 11 with a mean of 6.0 ± 2.4 eggs/column and differed significantly between columns (χ^2^ = 111.380, df = 9, *P* < 0.001). Significant differences were found between columns 2 and 1, 7, 8; 3 and 1, 6, 7, 8; and 4 and 1, 6, 7, 8 (**Figure 3C**). A significant positive correlation was found between column number and egg number for the egg masses (F = 84.820, df = 1, 90, *P* < 0.001) (**Figure 3D**), with total eggs in each egg mass increasing with the increase of columns in it.

### Egg mass size

**Figure 4:**
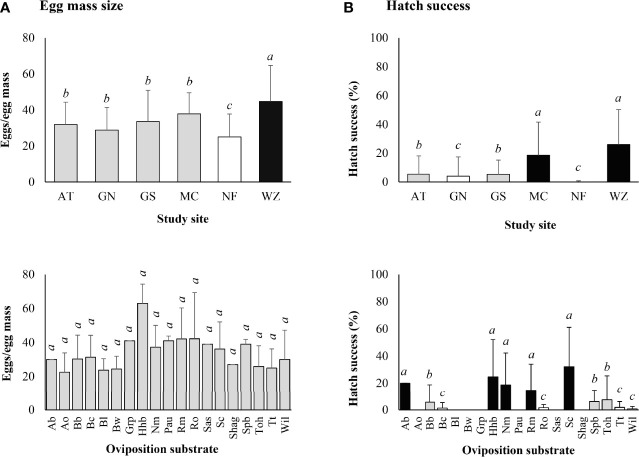
*Lycorma delicatula*. **(A)** Egg mass size and **(B)** egg hatch success by study site and type of oviposition substrate based on laboratory observations in 2019. AT—Antietam, GN—Gibraltar North, GS—Gibraltar South, MC—Marsh Creek, NF—Nolde Forest, WZ—Wertz. Ab—American beech, Ao—autumn olive, Bb—black birch, Bc—black cherry, Bl—black locust, Bw—black walnut, Grp—summer grape, Hhb—American hophornbeam, Nm—Norway maple, Pau—princess tree, Rm—red maple, Ro—red oak, Sas—Sassafras, Sc—sweet cherry, Shag—shagbark hickory, Spb—northern spicebush, Toh—tree-of-heaven, Tt—tuliptree, Wil—black willow. Means with the same lowercase letters are not significantly different (A-negative binomial generalized model, B-generalized linear model with binomial distribution, α = 0.05).

The mean (± SD) egg mass size was 33.7 ± 16.0 eggs/egg mass for the 300 egg masses collected in 2019. Significantly larger egg masses were found at Wertz (Z-value = 3.624, *P* < 0.001) and smaller ones at Nolde Forest (Z-value = -2.619, *P* = 0.009) (**Figure 4A**). No significant difference in egg mass size was found between different types of oviposition substrates (α = 0.05) (**Figure 4A**).

**Figure 5:**
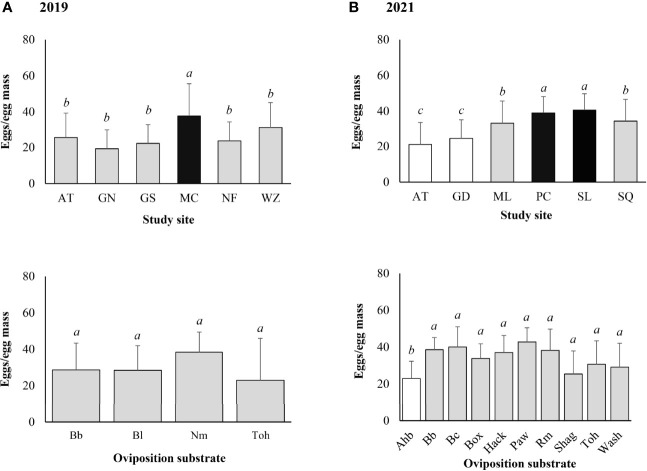
*Lycorma delicatula* egg mass size by study site and type of oviposition substrate based on field monitoring in **(A)** 2019 and **(B)** 2021. AT—Antietam, GD—Gordon, GN—Gibraltar North, GS—Gibraltar South, MC—Marsh Creek, ML—Memorial Lake, NF—Nolde Forest, PC—Pinnacle, SL—Sam Lewis, SQ—Susquehannock, WZ—Wertz. Ahb—American hornbeam, Bb—black birch, Bc—black cherry, Bl—black locust, Box—boxelder, Hack—hackberry, Nm—Norway maple, Paw—pawpaw, Rm—red maple, Shag—shagbark hickory, Toh—tree-of-heaven, Wash—white ash. Means with the same lowercase letters are not significantly different (negative binomial generalized model, α = 0.05).

The mean (± SD) egg mass size was 26.6 ± 14.5 eggs/egg mass for the 120 egg masses marked in the field in 2019. Significantly larger egg masses were found at Marsh Creek (Z-value = 2.304, *P* = 0.021). No significant difference in egg mass size was found between different types of oviposition substrates (α = 0.05) (**Figure 5A**).

The mean (± SD) egg mass size was 35.1 ± 11.9 eggs/egg mass for the 92 egg masses marked in the field in 2021. Significantly larger egg masses were found at Sam Lewis (Z-value = 3.275, *P* = 0.001) and Pinnacle (Z-value = 3.080, *P* = 0.002). Larger egg masses were also found at Susquehannock (Z-value = 2.460, *P* = 0.014) and Memorial Lake (Z-value = 2.283, *P* = 0.022) (**Figure 5B**). Significantly smaller egg masses were found on American hornbeam (Z-value = -2.023, *P* = 0.043) compared with other types of oviposition substrates (**Figure 5B**).

### Egg hatch in the laboratory

The three egg masses with no eggs were excluded from laboratory hatch study. Egg hatch started within 2 weeks after incubation and lasted for about 2 weeks for individual egg masses, with peak hatch occurring in the middle of the period. Only 39.7% of the egg masses contained at least one hatched egg in 2019, with the highest egg mass hatch success of 86% at Wertz, followed by Marsh Creek (64%), Gibraltar South (42%), Antietam (28%), Gibraltar North (14.3%), and Nolde Forest (2.1%). The mean (± SD) egg hatch success was 10.0 ± 18.5 (0–90)% for the egg masses, with a positive correlation between hatch success and egg mass size (Z-value = 15.880, *P* < 0.001).

Significantly higher egg hatch success was found at Wertz (Z-value = 14.967, *P* < 0.001) and Marsh Creek (Z-value = 10.856, *P* < 0.001) while significantly lower hatch success was found at Nolde Forest (Z-value = -5.801, *P* < 0.001) and Gibraltar North (Z-value = -2.063, *P* = 0.039) (**Figure 4B**).

Significant differences in egg hatch success were also observed between types of oviposition substrates, with lower rates on red oak (Z-value = -4.353, *P*<0.001), black cherry (Z-value = -4.262, *P* < 0.001), tuliptree (Z-value = -4.085, *P* < 0.001), black willow (Z-value = -3.852, *P* < 0.001), black birch (Z-value = -2.627, *P* = 0.009), tree-of-heaven (Z-value = -2.094, *P* = 0.036), and northern spicebush (Z-value = -1.993, *P* = 0.046) (**Figure 4B**). No eggs on autumn olive, black locust, black walnut, princess tree, sassafras, shagbark hickory, and summer grape hatched successfully (**Figure 4B**).

### Egg hatch in the field 2019

In 2019, *L. delicatula* egg hatch was first observed at Marsh Creek on 21 May, followed by Gibraltar South, Nolde Forest, Wertz, Gibraltar North, and Antietam in the following days in the field. Hatch generally completed within 2–3 weeks for individual egg masses, with the last egg hatch observed on 1 July at Nolde Forest. Overall, 69.2% of the egg masses contained at least one hatched egg, with the highest egg mass hatch success of 90% at Marsh Creek, followed by Gibraltar South (75%), Gibraltar North (70%), Nolde Forest (70%), Antietam (55%), and Wertz (55%). The mean (± SD) egg hatch success was 31.6 ± 30.9 (0–100)% for the egg masses, with a positive correlation between hatch success and egg mass size (Z-value = 4.478, *P* < 0.001).

**Figure 6:**
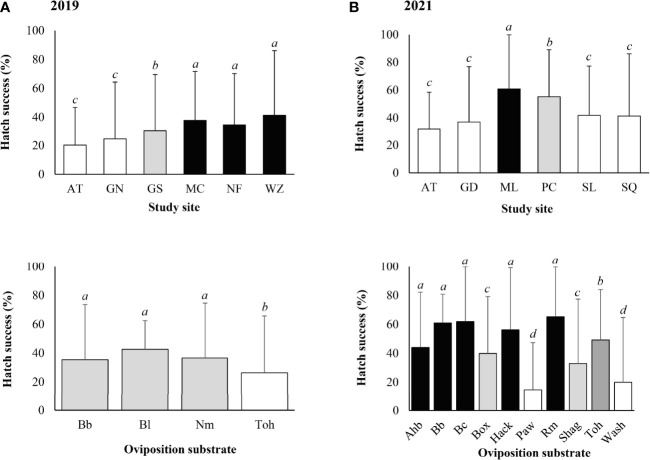
*Lycorma delicatula* egg hatch success by study site and type of oviposition substrate based on field monitoring in **(A)** 2019, **(B)** 2021. AT—Antietam, GD—Gordon, GN—Gibraltar North, GS—Gibraltar South, MC—Marsh Creek, ML—Memorial Lake, NF—Nolde Forest, PC—Pinnacle, SL—Sam Lewis, SQ—Susquehannock, WZ—Wertz. Ahb—American hornbeam, Bb—black birch, Bc—black cherry, Bl—black locust, Box—boxelder, Hack—hackberry, Nm—Norway maple, Paw—pawpaw, Rm—red maple, Shag—Shagbark hickory, Toh—tree-of-heaven, Wash—white ash. Means with the same lowercase letters are not significantly different (generalized linear model with binomial distribution, α = 0.05).

Significantly higher egg hatch success was found at Wertz (Z-value = 8.447, *P* < 0.001), Nolde Forest (Z-value = 5.220, *P* < 0.001), Marsh Creek (Z-value = 3.784, *P* < 0.001), and Gibraltar South (Z-value = 2.374, *P* = 0.018). Significantly lower egg hatch success was found on tree-of-heaven (Z-value = - 8.187, *P* < 0.001) compared with other types of oviposition substrates (**Figure 6A**).

### Egg hatch in the field 2021

*Lycorma delicatula* egg hatch in the field in 2021 was first observed at Pinnacle and Susquehannock on 24 May, followed by Memorial Lake, Sam Lewis, Antietam, and Gordon. Hatch generally completed within 2 weeks for individual egg masses, with the last egg hatch observed on 24 June at Gordon. Overall, 69.6% of the egg masses contained at least one hatched egg, with the highest egg mass hatch success of 80% at Pinnacle, followed by Antietam (75%), Memorial Lake (75%), Sam Lewis (75%), Gordon (62.5%), and Susquehannock (50%). The mean (± SD) egg hatch success was 48.0 ± 38.7 (0–100)% for the egg masses, with a positive correlation between hatch success and egg mass size (Z-value = 3.672, *P* < 0.001).

Significantly higher egg hatch success was found at Memorial Lake (Z-value = 4.277, *P* < 0.001) and Pinnacle (Z-value = 2.962, *P* = 0.003). Significantly lower hatch success was found on pawpaw (Z-value = -11.296, *P* < 0.001), white ash (Z-value = -7.417, *P* < 0.001), shagbark hickory (Z-value = -3.161, *P* = 0.002), boxelder (Z-value = -3.151, *P* = 0.002), and tree-of-heaven (Z-value = -2.425, *P* = 0.015) compared with other types of oviposition substrates (**Figure 6B**).

## Discussion

While oviposition substrate played an important role in *L. delicatula* egg mass structure and hatchability (16), more focus should probably be on habitat structure as those with more tree-of-heaven trees generally supported larger egg masses with more successful egg hatch (**Table 1**, **Figures 4**–**6**). This kind of oviposition selection can be explained by the proximity to suitable habitat for offspring hypothesis (25). The ability to feed on a wild range of hosts and to disperse freely between different host species (16, 26) makes young *L. delicatula* nymphs nearly independent of the oviposition substrates, rendering the preference–performance hypothesis (27) unlikely as long as tree-of-heaven is available in the habitat for necessary nutrition acquisition and defense sequestration (28). On the other hand, optimal foraging theory (29) should also be explored to shed light on the selective patterns on some tree species (e.g., maples) by late-stage adults as both feeding hosts and oviposition substrates (30, 31). Impacts of habitat and oviposition substrate on egg mass size have also been reported for parallel-banded leafroller moth (*Choristoneura parallela* (Robinson) [Lepidoptera: Tortricidae]) (32) and beet armyworm (*Spodoptera exigua* (Hübner) [Lepidoptera: Noctuidae]) (33).

Empty egg masses have been recorded for *L. delicatula* in a previous study (16). It is not yet clear how females decide to place a certain number of eggs in each column in the egg mass, and why no eggs are laid under the waxy cover in a few of them. In addition to low tree-of-heaven density in the habitats, chemical control of *L. delicatula* on tree-of-heaven in adjacent areas in the previous year might have a negative impact on the mean egg mass size at Nolde Forest in 2019, whereas a longer infestation history could have contributed to the smaller egg masses observed at Antietam and Gordon in 2021 (**Table 1**, **Figures 4**, **5**). However, the potential impact of herbicide treatment of tree-of-heaven in adjacent areas at Pinnacle and Susquehannock in 2021 still needs to be examined (**Table 1**, **Figure 5**). The largest egg mass ever recorded contained 192 eggs (16). Comparable egg mass sizes (30–50 eggs/egg mass) were also reported before (11, 13, 16, 20, 34).

In general, egg hatch was less successful in the laboratory compared with that in the field (**Figures 4**,**6**). However, this may change as laboratory rearing conditions improve in the near future. An egg hatch success of 20.5% was reported in the laboratory compared with 68.2% observed in the field in 2017 (16). In another study, 65.9% egg masses and 58.4% eggs hatched successfully at 15°C in the laboratory (21). On the other hand, egg hatch success dropped to 10.8% when held at 20°C constantly (21). A higher relative humidity and lower than 20°C incubation temperature in the laboratory may be needed to simulate field conditions in late May in southeastern Pennsylvania.

A difference in egg hatch success on different types of oviposition substrates has been reported before. About 80% of eggs on tree-of-heaven hatched whereas only 2%–3% of eggs on Japanese pagoda tree (*Styphnolobium japonicum* (L.) Schott [Fabales: Fabaceae]) and elms (*Ulmus* spp. [Rosales: Ulmaceae]) hatched successfully in the field in China (11). On the contrary, only 23% of eggs from tree-of-heaven hatched, whereas 79.6% of eggs from black locust hatched after 2 months of incubation in the laboratory in the United States (16). In Japan, egg hatch success was significantly reduced when wax cover was removed from the surface in the field (20). Oviposition substrates, waxy cover, collection disturbance, incubation conditions, and number of egg masses evaluated all contributed to the reported egg hatch success in the laboratory and the field (11, 16, 18–21).

Oviposition is a critical aspect of the reproductive biology for insects. The decision of when and where and the process of how to lay the eggs have profound impact on the fitness of the species (35). Habitat structure, site conditions, host availability and quality, inter- and intraspecific competition, parasitoids, and predators all play a role in oviposition site selection (1, 4, 36–41). Reproductive success also depends on optimal allocation of available resources by females toward quantity (large clutch size) or quality (large eggs) (42). *Lycorma delicatula* eggs are relatively well protected from adverse abiotic conditions with thick eggshells and wax cover. No predators rely solely on them while only two species of parasitoids are recorded in the field (43–45). Habitat suitability, tree-of-heaven density, substrate conditions, and intraspecific competition should be the most important factors in oviposition selection for *L. delicatula*.

Information from this study is beneficial to the understanding of *L. delicatula* population dynamics and its management in the field in North America. Infestations usually start in suitable habitats with tree-of-heaven trees (11). Egg masses are mostly found on tree-of-heaven and a few neighboring species in the habitats at the beginning (16). Onsite chemical control of *L. delicatula*, herbicide treatment of tree-of-heaven, and fungal epizootics could interfere with egg mass size and hatchability. Management strategies should therefore focus on newly infested tree-of-heaven trees with egg mass survey extended to preferred substrates in the habitats. Hatch success in the field should be used to evaluate current-generation nymphal populations since those measured in the laboratory were generally lower and more variable depending on incubation conditions. The impact of other key factors (tree-of-heaven health, climatic conditions, natural enemies, and management activities) in *L. delicatula* population dynamics based on egg mass evaluation should be investigated.

## Data availability statement

The original contributions presented in the study are included in the article/supplementary material. Further inquiries can be directed to the corresponding author.

## Author contributions

The author conceptualized the study; conducted the field work; collected and analyzed the data; wrote the draft; and reviewed and edited the final version of the manuscript.

## Funding

This project was supported by funding from the Pennsylvania Department of Conservation and Natural Resources.

## Acknowledgments

I thank Matthew Hunter for field assistance; Richard Hartlieb, Brent Erb, James Wassell, Brendan Lederer, Nur Ritter, Jennifer Chandler, Courtney Troutman, Nathaniel Brown, and Andrew Leidich for study site access. Comments by the reviewers improved the manuscript.

## Conflict of interest

The author declares that the research was conducted in the absence of any commercial or financial relationships that could be construed as a potential conflict of interest.

## Publisher’s note

All claims expressed in this article are solely those of the authors and do not necessarily represent those of their affiliated organizations, or those of the publisher, the editors and the reviewers. Any product that may be evaluated in this article, or claim that may be made by its manufacturer, is not guaranteed or endorsed by the publisher.
